# Mutation screening of the *RNF8, UBC13 *and *MMS2 *genes in Northern Finnish breast cancer families

**DOI:** 10.1186/1471-2350-12-98

**Published:** 2011-07-21

**Authors:** Mikko Vuorela, Katri Pylkäs, Robert Winqvist

**Affiliations:** 1 Laboratory of Cancer Genetics, Department of Clinical Genetics and Biocenter Oulu, University of Oulu, Oulu University Hospital, P.O. Box 5000, FI-90014, Oulu, Finland

## Abstract

**Background:**

Currently known susceptibility genes such as *BRCA1 *and *BRCA2 *explain less than 25% of familial aggregation of breast cancer, which suggests the involvement of additional susceptibility genes. RNF8, UBC13 and MMS2 are involved in the DNA damage response pathway and play important roles in BRCA1-mediated DNA damage recognition. Based on the evidence that several players in the ubiquitin-mediated BRCA1-dependent DDR seem to contribute to breast cancer predisposition, *RNF8, UBC13 *and *MMS2 *were considered plausible candidate genes for susceptibility to breast cancer.

**Methods:**

The entire coding region and splice junctions of *RNF8, UBC13 *and *MMS2 *genes were screened for mutations in affected index cases from 123 Northern Finnish breast cancer families by using conformation sensitive gel electrophoresis, high resolution melting (HRM) analysis and direct sequencing.

**Results:**

Mutation analysis revealed several changes in *RNF8 *and *UBC13*, whereas no aberrations were observed in *MMS2*. None of the found sequence changes appeared to associate with breast cancer susceptibility.

**Conclusions:**

The present data suggest that mutations in *RNF8, UBC13 *and *MMS2 *genes unlikely make any sizeable contribution to breast cancer predisposition in Northern Finland.

## Background

Breast cancer is the most frequent malignancy among women [1], and the presence of a family history is one of the most fundamental risk factors for the disease [2]. Currently known susceptibility genes including *BRCA1, BRCA2, ATM, CHEK2, PALB2, RAD51C *and *BRIP1 *explain less than 25% of familial breast cancer. The rest of the cases could be explained by mutations in mainly moderate and low penetrance cancer susceptibility genes together with environmental factors. Many of the genes already associated with breast cancer susceptibility encode proteins that operate together with BRCA1 and BRCA2 in the DNA damage response pathway (DDR) [3-7]. Other genes with similar functions thus represent good candidates for being new susceptibility genes.

Recent evidence indicates ubiquitin chain formation, recognition and breakdown at the site of DNA double-strand breaks (DSB) as an essential component of the DDR [8]. RNF8 is a RING-finger ubiquitin ligase (E3), which is recruited to the sites of DNA damage after ATM/ATR-dependent phoshorylation of the H2AX histone variant [9-11]. Together with its ubiquitin-conjugating enzyme (E2) partner UBC13 it mediates K63-linked polyubiquitin conjugation to histones H2A and H2AX. The RNF8/UBC13-dependent histone ubiquitylation is then amplified by the RNF168 E3-ligase acting in concert with UBC13 [12]. Ubiquitynated histones are recognized by RAP80 through its ubiquitin interaction motifs (UIMs), which provide an ubiquitin recognition element to target the BRCA1 E3 ligase, Abraxas, MERIT40, BRCC45 and a K63-ubiquitin specific deubiquitinating enzyme BRCC36 to DSBs. Each of these activities is required for appropriate checkpoint and repair responses to ionizing radiation [13-15]. Depletion of RNF8 or UBC13 *in vitro *leads to inhibition of the recruitment of 53BP1, BRCA1, RAP80 and Abraxas to DSB sites [9-11,16]. It has also been demonstrated that the depletion of RNF8 leads to increased ionizing radiation sensitivity and defective G2/M checkpoint [9-11]. In addition, *Rnf8^-/- ^*mice display increased genomic instability and higher risk for tumorigenesis, proposing that RNF8 is a novel tumor suppressor [17].

Besides RNF8, the ubiquitin E2 variant (UEV) MMS2 seems to be essential for certain functions of UBC13. MMS2 forms a complex with UBC13 [18], and this heterodimer formation has been demonstrated to be essential for the DNA damage repair function of UBC13 [19]. Suppression of UBC13 or MMS2 has been shown to increase the sensitivity to DNA damaging agents [19], although the exact role of MMS2 in DDR is still unclear [20].

We have previously reported a potentially deleterious germline variant of *RAP80 *(del81E) that abrogated ubiquitin binding and DNA damage response in breast cancer cases [21]. Additionally, recent findings of an extensive genome-wide linkage consortium study suggested an association between the rare allele of single nucleotide polymorphism (SNP) rs8170 in *MERIT40 *and an increased propensity for hormone receptor-negative breast cancer, both in the general population and in *BRCA1 *mutation carriers [22]. Based on the evidence that several players in the ubiquitin-mediated BRCA1-dependent DDR seem to contribute to breast cancer predisposition (summarized in Table 1), we decided to examine the role of *RNF8, UBC13 *and *MMS2 *in familial breast cancer by performing a comprehensive mutation screening of these genes in 123 Northern Finnish breast cancer families.

**Table 1:** Key operators in the BRCA1-dependent ubiquitin -mediated DNA damage recognition pathway and their currently known role in breast cancer predisposition

Gene	Previous studies on the role in breast cancer predisposition	Disease related alterations
*RNF8*	Not done	-
*RNF168*	Not done ^a^	-
*UBC13*	Not done	
*RAP80*	Mutation screening [21,27-29]	del81E [21]
*Abraxas*	Mutation screening [27,29]	N. I.
*MERIT40*	Mutation screening [30] GWAS [22]	N. I. rs8170 [22]
*BRCC45*	Not done	-
*BRCC36*	Not done	-

^a ^Homozygous mutations in this gene have been demonstrate to result in RIDDLE syndrome [12]

GWAS, genome wide association study; N. I., not identified

## Methods

### Study population

Affected index cases of 123 breast cancer families from Northern Finland were screened for germline mutations in the *RNF8, UBC13 *and *MMS2 *genes. From the studied families, 77 were classified as high-risk ones, defined as follows: 1) three or more cases of breast and/or ovarian cancer in first or second-degree relatives (median age 49 years, variation 37-80 years), or 2) two cases of breast cancer in first- or second-degree relatives, of which at least one with early disease onset (age ≤ 35 years), bilateral disease or multiple primary tumors. Most of the high-risk families presented three or more cancer cases. The remaining 46 families with moderate disease susceptibility indicated two cases of breast cancer in first- or second-degree relatives. Fourteen of the studied index cases had previously been tested positive for known breast cancer-associated germline mutations in *BRCA1 *or *BRCA2 *(eleven) and *PALB2 *(three). DNA samples from anonymous cancer-free female individuals obtained from Finnish Red-Cross blood donors (N = 104-299, depending on the tested mutation), originating from the same geographical region as the studied cancer cases, were used as controls. All patients had given their informed consent for acquisition of pedigree data and blood specimens for the study of cancer susceptibility gene mutations. Approval to perform the study was obtained from the Ethical Board of the Northern Ostrobothnia Health Care District and the Finnish Ministry of Social Affairs and Health.

### DNA extraction and mutation analysis

Genomic DNA was extracted from blood lymphocytes using either the standard phenol-chloroform method or the Puregene D-50K purification kit (Gentra, Minneapolis, MN, USA). Mutation screening of the coding regions and exon-intron boundaries of the *RNF8, UBC13 *and *MMS2 *genes was carried out by conformation sensitive gel electrophoresis (CSGE) [23], high resolution melting (HRM) analysis [24], or by direct sequencing. Samples with band shifts in CSGE or deviant melt curves in HRM were reamplified and sequenced with Li-Cor IR2 4200-S DNA Analysis system (Li-Cor, Inc., Lincoln, NE, USA) or with capillary sequencing using ABI3130xl Genetic Analyzer (Applied Biosystems, Foster City, CA, USA). For Li-Cor the Sequi Therm EXEL II DNA Sequencing Kit-LC (Epicentre Technologies, Madison, WI, USA) and for ABI the Big dye terminator kit v1.1 (Applied Biosystems, Foster City, CA, USA) were used. Chromatograms were interpreted using CodonCode Aligner v. 3.5.4 (Codon Code Corporation, Dedham, MA, USA) and with MEGA4 [25]. Oligonucleotides for CSGE, HRM and sequencing (Table 2) were designed using Primer3 software [26], based on sequence information obtained from public databases (NC_000006.11, NC_000012.11 and NC_000008.10).

**Table 2:** Primers used for the screening of *RNF8, UBC13 *and *MMS2*

Gene	Exon	Forward primer (5'-3')	Reverse primer (5'-3')	PCR fragment size (bp)
*RNF8*	1	GCGAGGAGACCTCTCGAATC	TCCTCTCTGCCATTCATTCA	498
	2	TGCTGCTGGTTGATGAGAT	AAATAAAAGTCATTAGGCTTCTG	250
	3a	AAGAAGACGAAAATCATGAAGC	TAATTCATCCAAACTGAATTTCC	294
	3b	CCTTGTCTTTCCCCAAAGAAT	TTACTTGGCTCAAGGGCAGT	242
	3c	AGTGGCCAGTACACCCTCTG	TTCACATTCATAACGGCTTCA	240
	3d	GGTGACCATGTCCAGGATTC	AAGACCACTCTTGCCCTTCC	260
	4	CAGGAGATTTTCCACCTGCT	GGTCATGTGATGCCTGTTTG	271
	5	CAGGCATGTTTGTGGCTAAA	CCTAGCAACCCTTGCACTGT	242
	6	CCTGTCCCATTTTGCATTTT	AAGGGGTGAGCAACTGTTC	197
	7	GCCCTTAAGATGGGATTGTTG	TCCCTTTACTCCTCCCCATT	483
	8	AGGGAAATACAGGCTCCTCA	CAAGTGACTGAGGGCTTCCT	220
*UBC13*	1	GACTTCCACTCGTGCGTGA	TCCTCAGCACCCGACTTC	264
	2	TTGGGAGATTGGAGCTGTTC	TGGAATGCTTAAGAGAAAAAGGA	430
	3	GTCTGTGGGAGGGAAGTGAA	CCCATAGCAAGCCATTTTGT	385
	4	ATCTTTCAGCCCTGATCCAA	GAGGGGCCACTGCTTTTA	448
*MMS2*	1	CCCGGCCCTCATGAACTT	GGTCCCAGGCTACGCTCT	411
	2	AGGGGATTTGGTCTTTTTGG	CACGTGGGAAGCATCAATAA	421
	3	GCACTTAGACATTAATATTTTAGGTA	TTTTGGCTTAACAAAGGTCCTC	331
	4	TGCTTAACAAATTGGTGCCATA	GCTGCATTTTTCCTCCTGTT	408

### Statistical and bioinformatical analysis

Carrier frequencies between patients and healthy controls were compared by using Pearson Chi-Square or Fisher's exact test in PASW Statistics (version 18 for Windows, SPSS Inc., Chicago, IL, USA), which was also used for the generation of odds ratios and confidence intervals. All alterations were checked with NNSplice software for potential splicing effects http://www.fruitfly.org/seq_tools/splice.html.

## Results

The mutation analysis of *RNF8 *revealed two exonic, two intronic and three 5'UTR variants. Only one of these variants was novel (not reported the NCBI SNP database, http://www.ncbi.nlm.nih.gov/SNP/). Both of the observed exonic variants of *RNF8 *were synonymous. In the *UBC13 *gene, one unreported and one known intronic variant were observed, whereas no sequence alterations were observed in *MMS2*. All observed alterations in *RNF8 *and *UBC13 *were assessed for possible effects on consensus splice sites, but none of them had a predicted effect on splicing. In order to evaluate possible pathogenicity of the observed changes, their frequencies were compared between cases and healthy control individuals. None of the found sequence changes, however, appeared to associate with breast cancer susceptibility (Table 3).

**Table 3:** Observed alterations in the *RNF8*,*UBC13 *and *MMS2 *genes in Finnish breast cancer families

Gene	Nucleotide change	rs number	Carrier frequency		P-value
				
			Familial cases	Controls	(OR; 95% CI)
*RNF8*	*RNF8*ex1-36 C > T (5'-UTR)	-	4.8% (6/123)	1.9% (2/104)	0.29 (2.6; 0.52-13.2)
	*RNF8*ex1-150 G > T (5'-UTR)	rs4714059	12.2% (15/123)	19.2% (20/104)	0.20 (0.58; 0.28-1.21)
	*RNF8*ex1-134 C > G (5'-UTR)	rs195420	22.8% (28/123)	18.3% (19/104)	0.42 (1.32; 0.69-2.53)
	*RNF8*ex4+17 A > G (intron)	rs77440008	1.6% (2/123)	5.9% (16/273)	0.07 (0.27; 0.06-1.17)
	*RNF8*ex7-6 C > T (intron)	rs2284923	41.5% (51/123)	46.7% (121/259)	0.38 (0.81; 0.52-1.25)
	*RNF8*ex7 G1344A (Thr448Thr)	rs2284922	36.6% (45/123)	41.1% (111/270)	0.44 (0.83; 0.53-1.28)
	*RNF8*ex7 G1377A (Lys459Lys)	rs34150698	17.9% (22/123)	19.3% (52/270)	0.78 (0.91; 0.53-1.59)
					
*UBC13*	*UBC13*ex3+17C > T (intron)	rs7969431	3.3% (4/123)	4.7% (14/299)	0.61 (0.68; 0.22-2.12)
	*UBC13*ex4-18 G > T (intron)	-	1.6% (2/123)	3.4% (10/297)	0.52 (0.47; 0.10-2.20)
*MMS2*	-	-	-	-	-

OR, Odds ratio; CI, confidence interval; UTR, untranslated region

## Discussion

RNF8, UBC13 and MMS2 have important roles in the maintenance of genomic integrity and cell-cycle checkpoint control [9-11,19]. Based on their involvement in DDR and importance in BRCA1-mediated DNA damage recognition it was considered possible that mutations in these genes might contribute to hereditary predisposition to breast cancer.

In the current study, the whole coding region of the *RNF8, UBC13 *and *MMS2 *genes was systematically screened for mutations in 123 breast cancer families. No deleterious sequence alterations were observed in any of the genes. Previous studies have suggested that the *RNF8 *gene could be novel tumor suppressor [17], but it seems that germline mutations predisposing to breast cancer in this gene do not exist or, at least, are very rare. It is of interest that another E3 ligase, RNF168, which acts together with UBC13 to amplify the RNF8-dependent histone ubiquitylation has been shown to be defected in RIDDLE syndrome, which is an immunodeficiency and radiosensitivity disorder. However, it is still unclear whether RIDDLE syndrome is associated with genome instability or increased tumor incidence [12].

## Conclusion

The present data suggest that mutations predisposing to breast cancer are either very rare or absent in the coding region of the *RNF8, UBC13 *and *MMS2 *genes, which could possibly point to the essentiality of their protein products in the DNA damage response and other functions maintaining genomic integrity. Although a small study like this cannot exclude the possibility of some other rare mutations in *RNF8, UBC13 *and *MMS2 *might predispose to breast cancer, based on our findings they unlikely make any sizeable contribution to cancer predisposition. To our knowledge, this is the first study reporting the mutation screening of the *RNF8, UBC13 *and *MMS2 *genes in familial breast cancer cases.

## Competing interests

The authors declare that they have no competing interests.

## Authors' contributions

MV carried out the mutation screening and data analysis, and drafted the manuscript. RW and KP designed the study and revised the manuscript. All authors read and approved the final manuscript.

## Pre-publication history

The pre-publication history for this paper can be accessed here:

http://www.biomedcentral.com/1471-2350/12/98/prepub

## References

[B1] Parkin DM Bray F Ferlay J Pisani P Global cancer statistics, 2002 CA Cancer J Clin 2005 55 2 74 108 10.3322/canjclin.55.2.74 15761078

[B2] Collaborative Group on Hormonal Factors in Breast Cancer Familial breast cancer: collaborative reanalysis of individual data from 52 epidemiological studies including 58,209 women with breast cancer and 101,986 women without the disease Lancet 2001 358 9291 1389 1399 11705483 10.1016/S0140-6736(01)06524-2

[B3] Antoniou AC Easton DF Models of genetic susceptibility to breast cancer Oncogene 2006 25 43 5898 5905 10.1038/sj.onc.1209879 16998504

[B4] Ripperger T Gadzicki D Meindl A Schlegelberger B Breast cancer susceptibility: current knowledge and implications for genetic counselling Eur J Hum Genet 2009 17 6 722 731 10.1038/ejhg.2008.212 19092773 PMC2947107

[B5] Erkko H Xia B Nikkilä J Schleutker J Syrjäkoski K Mannermaa A Kallioniemi A Pylkäs K Karppinen SM Rapakko K Miron A Sheng Q Li G Mattila H Bell DW Haber DA Grip M Reiman M Jukkola-Vuorinen A Mustonen A Kere J Aaltonen LA Kosma VM Kataja V Soini Y Drapkin RI Livingston DM Winqvist R A recurrent mutation in PALB2 in Finnish cancer families Nature 2007 446 7133 316 319 10.1038/nature05609 17287723

[B6] Meindl A Hellebrand H Wiek C Erven V Wappenschmidt B Niederacher D Freund M Lichtner P Hartmann L Schaal H Ramser J Honisch E Kubisch C Wichmann HE Kast K Deissler H Engel C Muller-Myhsok B Neveling K Kiechle M Mathew CG Schindler D Schmutzler RK Hanenberg H Germline mutations in breast and ovarian cancer pedigrees establish RAD51C as a human cancer susceptibility gene Nat Genet 2010 42 5 410 414 10.1038/ng.569 20400964

[B7] Seal S Thompson D Renwick A Elliott A Kelly P Barfoot R Chagtai T Jayatilake H Ahmed M Spanova K North B McGuffog L Evans DG Eccles D Breast Cancer Susceptibility Collaboration (UK) Easton DF Stratton MR Rahman N Truncating mutations in the Fanconi anemia J gene BRIP1 are low-penetrance breast cancer susceptibility alleles Nat Genet 2006 38 11 1239 1241 10.1038/ng1902 17033622

[B8] Al-Hakim A Escribano-Diaz C Landry MC O'Donnell L Panier S Szilard RK Durocher D The ubiquitous role of ubiquitin in the DNA damage response DNA Repair (Amst) 2010 9 12 1229 1240 10.1016/j.dnarep.2010.09.011 PMC7105183 21056014

[B9] Huen MS Grant R Manke I Minn K Yu X Yaffe MB Chen J RNF8 transduces the DNA-damage signal via histone ubiquitylation and checkpoint protein assembly Cell 2007 131 5 901 914 10.1016/j.cell.2007.09.041 18001825 PMC2149842

[B10] Kolas NK Chapman JR Nakada S Ylänkö J Chahwan R Sweeney FD Panier S Mendez M Wildenhain J Thomson TM Pelletier L Jackson SP Durocher D Orchestration of the DNA-damage response by the RNF8 ubiquitin ligase Science 2007 318 5856 1637 1640 10.1126/science.1150034 18006705 PMC2430610

[B11] Mailand N Bekker-Jensen S Faustrup H Melander F Bartek J Lukas C Lukas J RNF8 ubiquitylates histones at DNA double-strand breaks and promotes assembly of repair proteins Cell 2007 131 5 887 900 10.1016/j.cell.2007.09.040 18001824

[B12] Stewart GS Panier S Townsend K Al-Hakim AK Kolas NK Miller ES Nakada S Ylanko J Olivarius S Mendez M Oldreive C Wildenhain J Tagliaferro A Pelletier L Taubenheim N Durandy A Byrd PJ Stankovic T Taylor AM Durocher D The RIDDLE syndrome protein mediates a ubiquitin-dependent signaling cascade at sites of DNA damage Cell 2009 136 3 420 434 10.1016/j.cell.2008.12.042 19203578

[B13] Sobhian B Shao G Lilli DR Culhane AC Moreau LA Xia B Livingston DM Greenberg RA RAP80 targets BRCA1 to specific ubiquitin structures at DNA damage sites Science 2007 316 5828 1198 1202 10.1126/science.1139516 17525341 PMC2706583

[B14] Shao G Patterson-Fortin J Messick TE Feng D Shanbhag N Wang Y Greenberg RA MERIT40 controls BRCA1-Rap80 complex integrity and recruitment to DNA double-strand breaks Genes Dev 2009 23 6 740 754 10.1101/gad.1739609 19261746 PMC2661612

[B15] Shao G Lilli DR Patterson-Fortin J Coleman KA Morrissey DE Greenberg RA The Rap80-BRCC36 de-ubiquitinating enzyme complex antagonizes RNF8-Ubc13-dependent ubiquitination events at DNA double strand breaks Proc Natl Acad Sci USA 2009 106 9 3166 3171 10.1073/pnas.0807485106 19202061 PMC2651241

[B16] Wang B Elledge SJ Ubc13/Rnf8 ubiquitin ligases control foci formation of the Rap80/Abraxas/Brca1/Brcc36 complex in response to DNA damage Proc Natl Acad Sci USA 2007 104 52 20759 20763 10.1073/pnas.0710061104 18077395 PMC2410075

[B17] Li L Halaby MJ Hakem A Cardoso R El Ghamrasni S Harding S Chan N Bristow R Sanchez O Durocher D Hakem R Rnf8 deficiency impairs class switch recombination, spermatogenesis, and genomic integrity and predisposes for cancer J Exp Med 2010 207 5 983 997 10.1084/jem.20092437 20385750 PMC2867283

[B18] Hofmann RM Pickart CM Noncanonical MMS2-encoded ubiquitin-conjugating enzyme functions in assembly of novel polyubiquitin chains for DNA repair Cell 1999 96 5 645 653 10.1016/S0092-8674(00)80575-9 10089880

[B19] Andersen PL Zhou H Pastushok L Moraes T McKenna S Ziola B Ellison MJ Dixit VM Xiao W Distinct regulation of Ubc13 functions by the two ubiquitin-conjugating enzyme variants Mms2 and Uev1A J Cell Biol 2005 170 5 745 755 10.1083/jcb.200502113 16129784 PMC2171356

[B20] Huen MS Huang J Yuan J Yamamoto M Akira S Ashley C Xiao W Chen J Noncanonical E2 variant-independent function of UBC13 in promoting checkpoint protein assembly Mol Cell Biol 2008 28 19 6104 6112 10.1128/MCB.00987-08 18678647 PMC2547017

[B21] Nikkilä J Coleman KA Morrissey D Pylkäs K Erkko H Messick TE Karppinen SM Amelina A Winqvist R Greenberg RA Familial breast cancer screening reveals an alteration in the RAP80 UIM domain that impairs DNA damage response function Oncogene 2009 28 16 1843 1852 10.1038/onc.2009.33 19305427 PMC2692655

[B22] Antoniou AC Wang X Fredericksen ZS McGuffog L Tarrell R Sinilnikova OM Healey S Morrison J Kartsonaki C Lesnick T Ghoussaini M Barrowdale D EMBRACE Peock S Cook M Oliver C Frost D Eccles D Evans DG Eeles R Izatt L Chu C Douglas F Paterson J Stoppa-Lyonnet D Houdayer C Mazoyer S Giraud S Lasset C Remenieras A et al. A locus on 19p13 modifies risk of breast cancer in BRCA1 mutation carriers and is associated with hormone receptor-negative breast cancer in the general population Nat Genet 2010 42 10 885 892 10.1038/ng.669 20852631 PMC3130795

[B23] Körkkö J Annunen S Pihlajamaa T Prockop DJ Ala-Kokko L Conformation sensitive gel electrophoresis for simple and accurate detection of mutations: comparison with denaturing gradient gel electrophoresis and nucleotide sequencing Proc Natl Acad Sci USA 1998 95 4 1681 1685 10.1073/pnas.95.4.1681 9465076 PMC19147

[B24] Wittwer CT High-resolution DNA melting analysis: advancements and limitations Hum Mutat 2009 30 6 857 859 10.1002/humu.20951 19479960

[B25] Kumar S Nei M Dudley J Tamura K MEGA: a biologist-centric software for evolutionary analysis of DNA and protein sequences Brief Bioinform 2008 9 4 299 306 10.1093/bib/bbn017 18417537 PMC2562624

[B26] Rozen S Skaletsky H Primer3 on the WWW for general users and for biologist programmers Methods Mol Biol 2000 132 365 386 10547847 10.1385/1-59259-192-2:365

[B27] Novak DJ Sabbaghian N Maillet P Chappuis PO Foulkes WD Tischkowitz M Analysis of the genes coding for the BRCA1-interacting proteins, RAP80 and Abraxas (CCDC98), in high-risk, non-BRCA1/2, multiethnic breast cancer cases Breast Cancer Res Treat 2009 117 2 453 459 10.1007/s10549-008-0134-y 18695986

[B28] Akbari MR Ghadirian P Robidoux A Foumani M Sun Y Royer R Zandvakili I Lynch H Narod SA Germline RAP80 mutations and susceptibility to breast cancer Breast Cancer Res Treat 2009 113 2 377 381 10.1007/s10549-008-9938-z 18306035

[B29] Osorio A Barroso A García MJ Martínez-Delgado B Urioste M Benítez J Evaluation of the BRCA1 interacting genes RAP80 and CCDC98 in familial breast cancer susceptibility Breast Cancer Res Treat 2009 113 2 371 6 10.1007/s10549-008-9933-4 18270812

[B30] Solyom S Patterson-Fortin J Pylkäs K Greenberg RA Winqvist R Mutation screening of the MERIT40 gene encoding a novel BRCA1 and RAP80 interacting protein in breast cancer families Breast Cancer Res Treat 2010 120 1 165 168 10.1007/s10549-009-0453-7 19572197 PMC2863093

